# Cross-cultural adaptation, validity and reliability of the Chinese Version of Miller Behavioral Style Scale

**DOI:** 10.1186/s12955-021-01717-9

**Published:** 2021-03-16

**Authors:** Qiqi Zhuo, Changsheng Cui, Hongmin Liang, Yangjuan Bai, Qiulan Hu, Ardani Latifah Hanum, Mingfang Yang, Yanjiao Wang, Wei Wei, Lan Ding, Fang Ma

**Affiliations:** 1 Department of Nursing, The First Affiliated Hospital of Kunming Medical University, Xichang Road, Kunming, 295# Yunnan China; 2 Pharmacy Department, Zigong First People’s Hospital, Zigong, Sichuan China; 3 Cardiology Department, The First Affiliated Hospital of Kunming Medical University, Kunming, Yunnan China; 4 ICU in Geriatric Department, The First Affiliated Hospital of Kunming Medical University, Kunming, Yunnan China; 5 Urology Department, The First Affiliated Hospital of Kunming Medical University, Kunming, Yunnan China; 6 Psychiatric Department, The First Affiliated Hospital of Kunming Medical University, Kunming, Yunnan China; 7 General Surgery Department, The First Affiliated Hospital of Kunming Medical University, Kunming, Yunnan China; 8 Out-Patient Department, The First Affiliated Hospital of Kunming Medical University, Kunming, Yunnan China

**Keywords:** Health education, Reliability, Validity, Information-seeking styles

## Abstract

**Background:**

Health education basing on patients’ information-seeking styles can improve the effectiveness of health education and patients’ health outcomes. The Miller Behavioral Style Scale (MBSS) is widely used to identify individual’s information-seeking styles, but the Chinese version is lacking. The study aim was to translate and culturally adapt the MBSS into Chinese version and test the content validity, construct validity and internal consistency reliability of the Chinese version of MBSS (C-MBSS).

**Methods:**

The forward-back-translation procedure was adopted in the translation of the MBSS. Content validity was assessed in a panel of experts. In a sample of 1343 individuals including patients, patients’ caregivers, university students, and medical staff, reliability and construct validity were assessed using Cronbach’s alpha coefficient and factor analysis. The measurement invariance across samples was tested using multigroup confirmatory factor analysis (MGCFA). Floor and ceiling effects were checked.

**Results:**

The C-MBSS achieved conceptual and semantic equivalence with the original scale. The item-level content validity index (I-CVI) of each item ranged from 0.78 to 1, and the averaging scale-level content validity index (S-CVI/ Ave) was 0.95. The exploratory factor analysis resulted in 2-factor assumption for each hypothetical threat-evoking scenario. Confirmatory factor analysis demonstrated a good fit between theoretical model and data, which provided confirmatory evidence for the second-order factor structure of 2-factor solution (Monitoring and Blunting). The Cronbach's alpha coefficients for the Monitoring and Blunting sub-scales of the C-MBSS were 0.75 and 0.62 respectively. MGCFA results supported the measurement invariance for the Monitoring sub-scale of the C-MBSS across samples. No floor or ceiling effects occurred.

**Conclusions:**

This study indicates that the C-MBSS has good content and construct validity. The Monitoring sub-scale of the C-MBSS had acceptable internal consistency reliability while the Blunting sub-scale had unsatisfactory one, which suggest that the Monitoring sub-scale of the C-MBSS can be used to identify individuals’ information-seeking styles in Chinese contexts across different populations.

## Background

Although several studies have reported that when confronted with medical stressors, people prefer high levels of health-related information in medical contexts and fare better when it is provided [1–3]. However, it has also been evidenced that sometimes the wealth of health-related information can be as dangerous as it is helpful [4–7]. Deyirmenjian et al. reported that for open-heart patients, the ones with more information providence showed higher levels of preoperative and postoperative anxiety compared with patients almost with no information giving [8]. Montazeri et al. found out that after giving cancer-related information to women in a waiting room at the breast cancer center, they became upset and anxiety [9]. Miller et al. mentioned that when women were exposed to cervical cancer risk information, they might catastrophize health dangers and felt intensely anxious and vulnerable [10]. All above suggest that not all persons desire information and the information-seeking styles of individuals should be taken into consideration in the information providence process.

Miller proposed the “Blunting Hypothesis” based on Seligman's safety signal theory, which accounts for individual differences about information- seeking styles [11]. In this hypothesis, individuals were categorized into two different information-seeking styles in seeking, encoding, processing and managing health-relevant risk and disease information: monitoring information-seeking styles (monitoring or monitors) and blunting information-seeking styles (blunting or blunters) [11, 12]. Monitors typically scan the environment for health threat-relevant information and amplify the threats cognitively, whereas blunters cope with aversive health events by distraction. In health threat situations, monitors prefer detailed health-related information and fare better when it is given, and they tend to perceive more risks and show great anxiety or distress when information is not readily available [1, 11]. On the contrary, blunters do better with less information and their anxiety may be increased when information is supplied too much [11]. Miller mentioned that when patients receive health information which matches their information-seeking styles, they have better outcomes psychologically, behaviorally and physiologically, therefore, patients’ information-seeking styles need to be taken into consideration before providing health care information [13–16]. The first step is to identify individual variations in information-seeking styles, which requires validated measures of individual information-seeking styles preferences.

There exist several scales that can predict information-seeking styles, such as Sentence Completion Test, Repression-Sensitization Scale and the Miller Behavioral Style Scale (MBSS) [12, 17, 18]. The Sentence Completion Test scale with 60 sentence stems is structurally complex and has limited validity and unsatisfactory reliability. Furthermore, monitors do not respond well to the scale [17]. The same problems also exist with The Repression-Sensitization Scale, which consists of 156 scorable and 26 buffer items and has low reliability in predicting information-seeking styles [11, 18]. The 32-item MBSS is a reliable and validated scale, and by far the most extensively used scale for predicting information-seeking styles [19–21]. It has been translated into different versions in different countries and widely used in different populations (children, student, general population, patients) to measure individual’s information-seeking styles in the face of threat-relevant information, which has shown good discriminant and convergent validity compared with other scales [11]. However, according to Rees’s [20] literature review about the psychometric properties of the MBSS scale, most studies were conducted in small sample sizes of students in western culture and the Cronbach’s alpha coefficient was rarely reported, whether it is applicable in Chinese culture needs further study. Therefore, this study aims to cross-culturally adapt the MBSS into Chinese and verify its internal consistency reliability, content validity and construct validity among individuals in Mainland China using a large sample size in medical and non-medical settings.

## Methods

### Design

A cross-sectional survey with a convenience sampling method was conducted from August to September 2019 in Yunnan Province, Southwest China. The MBSS can be used in different contexts (i.e., medical setting, worksite, academic context, community center, home setting), as well as across populations (i.e., individuals at-risk for disease, patients, and the healthy population). Our participants included university students, medical staff, patients receiving percutaneous coronary intervention and their caregivers, which could maximize the sample size and diversity. University students and medical staff got the Chinese version of MBSS (C-MBSS) via on line survey (wen juan xing). Patients receiving percutaneous coronary intervention and their caregivers were given 15 min to fill in the written C-MBSS in the hospital placement before their surgery. The research was approved by the ethics committee of the hospital.

### Measures

Miller Behavioral Style Scale (MBSS) is a commonly used 32-item measure to assess the information-seeking styles of individuals under threat. The scale consists of four hypothetical threat-evoking scenarios (1. fear of having dental work done; 2. kidnapped by a group of terrorist militants; 3. in danger of losing the job; 4. technical problems with the flight); each of which has eight corresponding potential coping responses, including 4 monitoring responses (e.g., "I would watch all the dentist's movements and listen for the sound of the drill") and 4 blunting responses (e.g., "I would try to think about pleasant memories"). There are totally 16 monitoring responses and 16 blunting responses, which constitute the Monitoring sub-scale and Blunting sub-scale respectively. With the permission of the original author of the scale, a 5-point Likert scale ranging from 1 “strongly unlikely” to 5 “strongly likely” was used for items scoring instead of the original dichotomous one, which allowed participants to give more varied and appropriate answers than dichotomous answers [20, 22]. The participants were asked to indicate the score that would apply to them for each response [12]. It is suggested by the original author that the Monitoring sub-scale and Blunting sub-scale can be used as separate scales and three scores can be derived from the C-MBSS questionnaire: (1) the total Monitoring sub-scale score ranging from 16 to 80; (2) the total Blunting sub-scale score ranging from 16 to 80; or (3) Monitoring minus Blunting ranging from 64 to − 64. Although individuals can be categorized into monitors or blunters by employing a median- or mean-split procedure on each of the three scores [20], it is the Monitoring sub-scale that is often used independently to identify individual’s information-seeking styles, with the score above the median being labeled as monitors and below the median score being labeled as blunters [12, 22]. Beyond that, demographic information including gender, age, nationality, occupation, educational background, diagnosis, type of operation etc. were also collected using a self-made demographic questionnaire.

### Translation procedure

With the written permission of the original author to use the MBSS, we translated the scale followed the forward-back-translation procedure [23]. Firstly, two native Chinese speakers with proficiency in English translated the scale into Mandarin Chinese. One translator is a nurse with master degree who studied in Ireland for one year; the other is an English linguistics scientist who had experiences of staying in London for two years. The two translated versions were selected and merged into a single version by the two translators. Secondly, two bilingual translators translated the Chinese version back to English. Translators were Chinese scholars who had worked in the USA for 10 years and were unaware of the research. The two English versions were selected and merged into a single version by the two translators. Semantic equivalence was conducted between the translation and the original version by an English native speaker. Thirdly, an expert committee composed of a psychiatrist, an English linguistics scientist and two nurses conducted cultural adaptation of the Chinese version to form a pre-final scale.

The readability and comprehensiveness of the scale were assessed in a convenience sample of 20 patients with coronary heart disease and 20 healthy university students. For the pilot test, after reading the four hypothetical stress-evoking scenarios of the C-MBSS, the participants were asked to tick the responses which they would most likely to do using a Likert 5-point scale (from strongly unlikely to strongly likely). Then a face-to-face interview was conducted to all participants to get their opinion about scale. According to the interview, all participants stated that they could understand the scenarios and responses easily, and it took approximately 10–15 min to finish the scale.

### Content validity

The item-level content validity index (I-CVI) and averaging scale-level content validity index (S-CVI/Ave) were used to evaluate the content validity [24]. Nine experts, including two psychological professors, one doctor, two advanced nurse practitioners and four associate professors in nursing, were invited to score and to evaluate item validity of the C-MBSS using clarity of phrasing and applicability of content as criteria [25]. Simultaneously, the experts gave suggestions on item modification and evaluated correlation level of each item for its corresponding construct using 4-point scale (from not relevant to highly relevant).

### Sample

More accurate solutions are achieved with larger sample sizes rather than the ratio of participants to variables of 1:5 to 1:10 [26]. Therefore, the item to participant ratio of 1:20 was used to calculate sample size. Considering possible data loss, we included more participants. Inclusion criteria were: (1) over 18 years old; (2) Having normal communication and literacy skills; (3) Voluntary participation. Participants with mental disorder or poor physical condition were excluded. Before the survey, participants received a brief introduction about the study and how to finish the questionnaire. All participants’ information was assured to keep confidentiality and verbal informed consent was obtained before data collection. A nursing graduate invited patients receiving percutaneous coronary intervention and their caregivers to fill in the written questionnaire face to face. There were 135 patients and 113 caregivers who met the inclusion criteria. Among them, 35 patients and 13 patients’ caregivers dropped out because of feeling troublesome; being transferred or deteriorated. Finally, 100 patients and 100 patients’ caregivers filled in the questionnaires respectively. A nursing manager invited the medical staff in a tertiary hospital to fill in the electronic questionnaire (wen juan xing) voluntarily and 550 medical staff filled in the questionnaires. A nursing teacher invited the students from grade 1 to grade 4 in a medical university to fill in the electronic questionnaire (wen juan xing) voluntarily and collected 750 questionnaires. Among the 1500 voluntary participants, 3 patients’ caregivers lost questionnaires, 43 medical staff and 52 students filled in the electronic questionnaire using less than 1 min and were automatically screened out by the system. Finally, the researchers collected 1402 returned questionnaires. The return rate of questionnaires was 93.47%. After screening, 59 questionnaires (10 questionnaires from patients, 16 questionnaires from medical staff and 33 questionnaires from students) that were not fully completed were excluded. Therefore, a total of 1343 valid questionnaires were returned for analysis; The valid return rate was 89.53%.

### Statistical analysis

Data base were established by Epidata 3.1 and then imported into Statistical Package for the Social Sciences version 20.0 (SPSS 20.0). Demographic data were analyzed using descriptive statistics. The validity and reliability of the C-MBSS were analyzed using SPSS 20.0 and Analysis of Moment Structure version 24 (AMOS 24). Firstly, factor analysis including exploratory factor analysis (EFA) and confirmatory factor analysis (CFA) were used to test the construct validity of the C**-**MBSS. The 1343 sample was divided into groups A and B randomly using SPSS 20.0. Sub-sample A (n = 672) was used for EFA. Kaiser–Meyer–Olkin (KMO) and Bartlett’s test of sphericity were used to test sampling adequacy and the suitability of data for factorisation respectively. Monte Carlo parallel analysis was used to extract factor number. Principal axis factoring with direct oblimin rotation was used to identify meaningful components [26, 27]. According to stevens’ advice [28], a sample size of 600 with a loading of 0.21 can be considered significant. Therefore, we deleted items with factor loading below 0.21 or cross-factor loading over 0.21. Sub-sample B (n = 671) was used for CFA to verify the factor structure of the C-MBSS derived from EFA. In the study, model fit was reflected by six fit indices including CMIN/DF, GFI, AGFI, CFI, RMSEA, SRMR [29]. Secondly, the Cronbach’s α coefficient was used to assess internal consistency reliability, and acceptable level should be greater than 0.7 [30]. Thirdly, MGCFA using the configural invariance, metric invariance and scalar invariance models was performed to verify the measurement invariance of the C-MBSS. The invariance model is considered acceptable when the value of CFI difference (ΔCFI) is below 0.010 [31]. Moreover, floor and ceiling effects were evaluated. 15% of participants achieving the highest or lowest score were considered as the threshold of significant ceiling or floor effects [30].

## Results

### Demographic characteristics

Of the 1343 participants who submitted eligible questionnaires, 655 (48.77%) were university students, 491 (36.56%) were medical staff, and 197 (14.67%) were patients receiving percutaneous coronary intervention and their caregivers. A total of 222 (16.53%) were male and 1121 (83.47%) were female. The age ranged from 18 to 82 years old with an average age of 27.97 years. 1074 (80%) were of Han nationality and 269 (20%) belonged to minority nationality. Among the participants, 65 (4.84%) had master degree or above, 1089 (81.09%) had baccalaureate degree, and 189 (14.07%) had associate degree or below.

### Content validity

According to nine experts’ responses and comments, the I-CVI of item1 and 18 were 0.67, and item 24, 25, 26 were 0.44, with experts’ comments of inappropriateness due to cultural diversity, which should be candidates for deletion [24]. Therefore, we deleted the five items with the value of I-CVI below 0.78. The I-CVI of remaining items ranged from 0.78 to 1 and the S-CVI/Ave was 0.95, indicating an adequate content validity of the 27-item version C-MBSS.

### Exploratory factor analysis

The C-MBSS is consisted of four hypothetical stress-evoking scenarios and theoretically the responses for each scenario are categorized into two factors, thus, we performed exploratory factor analysis to explore factor structures in each scenario. The Kaiser–Meyer–Olkin (KMO) for each scenario exceeded 0.5 and all Bartlett’s tests of sphericity were statistically significant (p < 0.001), which supported the use of factor analysis [32]. The parallel analysis resulted in 2-factor assumption for scenario 2, scenario 3 and scenario 4, and resulted in 3-factor assumption for scenario 1. According to the result of principal axis factoring and direct oblimin rotation, item 2 in scenario 1 was deleted because of factor loading lower than 0.21. The left 6 items in scenario 1 were re-performed EFA. The value of KMO and Bartlett’s tests of sphericity met target level. The parallel analysis resulted in 2-factor assumption for scenario 1 and the factor loadings of all items met requirements. The variance explained in each scenario ranged from 43.98% to 52.99%. Table 1 shows the rotated factor loadings of the item.

**Table 1:** Rotated factor loadings of the C-MBSS questionnaire items

Item	Factor loadings	Variance explained
**Scenario 1**		
**Factor 1: monitoring**		27.97
4. I would want the dentist to tell me when I would feel pain	0.33	
6. I would watch all the dentist's movements and listen for the sound of the drill	0.66	
7. I would watch the flow of water from my mouth to see if it contained blood	0.76	
**Factor 2: blunting**		25.02
3. I would try to think about pleasant memories	0.45	
5. I would try to sleep	0.54	
8. I would do mental puzzles in my mind	0.50	
**Scenario 2**		
**Factor 1: monitoring**		25.11
10. I would stay alert and try to keep myself from falling asleep	0.51	
12. If there was a radio present, I would stay near it and listen to the bulletins about what the police were doing	0.52	
13. I would watch every movement of my captors and keep an eye on their weapons	0.59	
16. I would make sure I knew where every possible exit was	0.52	
**Factor 2: blunting**		18.87
9. I would sit by myself and have as many daydreams and fantasies as I could	0.34	
11. I would exchange life stories with the other hostages	0.46	
14. I would try to sleep as much as possible	0.31	
15. I would think about how nice it's going to be when I get home	0.50	
**Scenario 3**		
**Factor 1: monitoring**		29.08
17. I would talk to my fellow workers to see if they knew anything about what the supervisor evaluation of me said	0.65	
20. I would try to remember any arguments or disagreements I might have had that would have resulted in the supervisor having a lower opinion of me	0.54	
23. I would try to think which employees in my department the supervisor might have thought had done the worst job	0.52	
**Factor 2: blunting**		21.37
19. I would go to the movies to take my mind off things	0.51	
21. I would push all thoughts of being laid off out of my mind	0.48	
22. I would tell my spouse that I'd rather not discuss my chances of being laid off	0.25	
**Scenario 4**		
**Factor 1: monitoring**		30.94
28. I would call for the flight attendant and ask what exactly the problem was	0.60	
30. I would listen carefully to the engines for unusual noises and would watch the crew to see if their behavior was out of the ordinary	0.46	
31. I would talk to the passenger beside me about what might be wrong	0.68	
**Factor 2: blunting**		20.96
27. I would watch the end of the movie, even if I had seen it before	0.47	
29. I would order a drink from the flight attendant or take a tranquilizer	0.38	
32. I would settle down and read a book or magazine or write a letter	0.44	

Bold indicates emphasize the titles and data of the scale and make them easy to be identified

### Confirmatory factor analysis

AMOS was used to construct a structural equation modelling with maximum likelihood to verify the 2-factor hypothesis in each scenario extracted from EFA. Table 2 presents the CFA fit indices for the four scenarios. These indices showed moderately good fit for the models and provided confirmatory evidence for the factor structure in the four scenarios [33, 34].

**Table 2:** Fit indices for confirmatory factor analysis of the four scenarios

Scenario	CMIN/DF	GFI	AGFI	CFI	RMSEA	SRMR
Scenario 1	2.775	0.989	0.972	0.968	0.051	0.0395
Scenario 2	5.466	0.962	0.927	0.810	0.082	0.0630
Scenario 3	6.329	0.976	0.937	0.829	0.089	0.0598
Scenario 4	3.588	0.986	0.964	0.920	0.062	0.0409

*CMIN/DF* chi-square/degrees of freedom, *GFI* goodness-of-fit index, *AGFI* adjusted goodness of fit index, *CFI* comparative fit index, *RMSEA* root mean square error of approximation, *SRMR* standardized root mean square residual

**Fig. 1:**
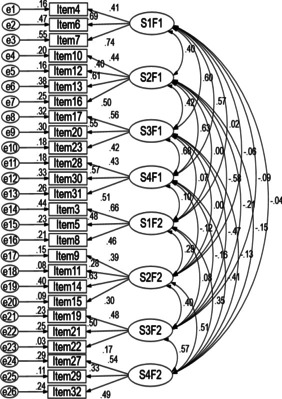
The correlations of the factors in four scenarios of the C-MBSS. *S1F1* scenario 1 factor 1, *S2F1* scenario 2 factor 1, *S3F1* scenario 3 factor 1, *S4F1* scenario 4 factor 1, *S1F2* scenario 1 factor 2, *S2F2* scenario 2 factor 2, *S3F2* scenario 3 factor 2, *S4F2* scenario 4 factor 2

**Fig. 2:**
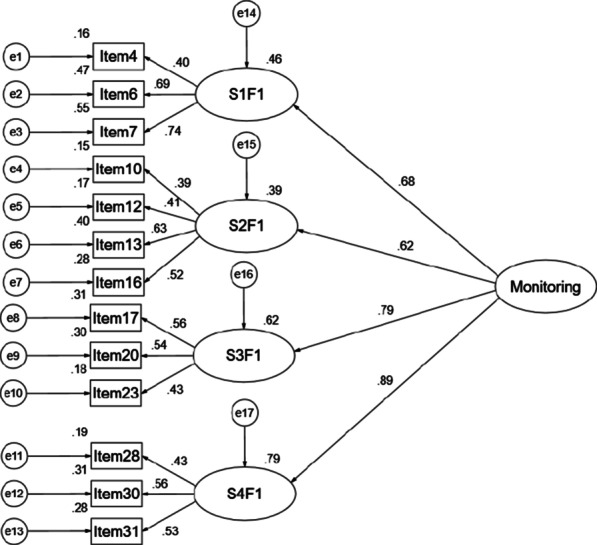
The second-order structural equation modelling of the factor structure of the Monitoring factor. *S1F1* scenario 1 factor 1, *S2F1* scenario 2 factor 1, *S3F1* scenario 3 factor 1, *S4F1* scenario 4 factor 1

**Fig.3:**
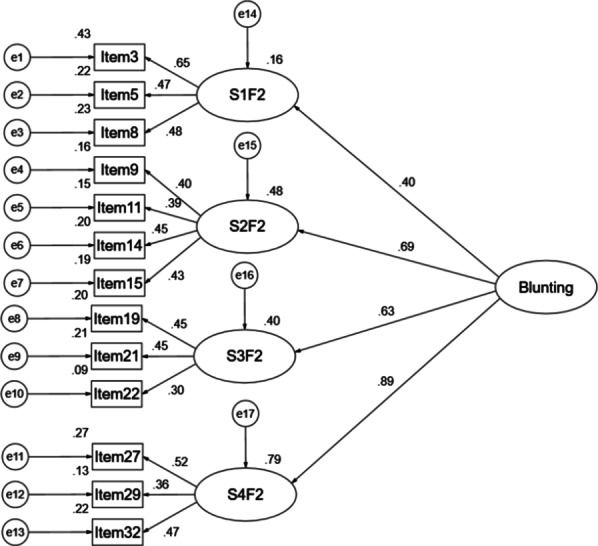
The second-order structural equation modelling of the factor structure of the Blunting factor. *S1F2* scenario 1 factor 2, *S2F2* scenario 2 factor 2, *S3F2* scenario 3 factor 2, *S4F2* scenario 4 factor 2

When we performed CFA, we found that factor 1 in scenario 1, factor 1 in scenario 2, factor 1 in scenario 3 and factor 1 in scenario 4 were strongly correlated, similarly, factor 2 in scenario 1, factor 2 in scenario 2, factor 2 in scenario 3 and factor 2 in scenario 4 were strongly correlated (seen in Fig. 1), which suggested that there existed second-order latent variables which might replace highly correlated factors to make the models more precise. Hence, we used the second-order CFA models to replace the first-order models. According to the research of Miller [11, 12], we assumed there were Monitoring and Blunting factors in the C-MBSS. The second-order models are shown in Figs. 2 and 3. In this study, the T values of Monitoring second-order CFA and Blunting second-order CFA were 0.94 and 0.97 respectively, which provided reasonable evidence of a second-order user satisfaction construct [35]. The model fit indices for Monitoring were: CMIN/DF = 2.253, GFI = 0.969, AGFI = 0.954, CFI = 0.931, RMSEA = 0.043, SRMR = 0.0429, and the model fit indices for Blunting were: CMIN/DF = 2.861, GFI = 0.962, AGFI = 0.943, CFI = 0.813, RMSEA = 0.053, SRMR = 0.0489, which indicated a good fit between theoretical model and data [33, 34, 36]. Compared with the model fits of the four scenarios, these values provided confirmatory evidence for the second-order factor structure.

### Internal consistency reliability

The results of internal consistency reliability tests showed that the Cronbach’s alpha coefficient of the Monitoring sub-scale and Blunting sub-scale of the C-MBSS were 0.75 and 0.62 respectively. The Cronbach’s alpha coefficient of the Blunting sub-scale was below acceptable limits [30].

### Measurement invariance

For the Monitoring factor model, all model fit indices reached the required standard in the four samples and the fit indices are shown in Table 3. The configural invariance model fit the data well (CMIN/DF = 1.800, GFI = 0.952, AGFI = 0.928, CFI = 0.920, RMSEA = 0.024, SRMR = 0.045). In addition, both the tests of metric invariance (ΔCFI = 0.009) and scalar invariance (ΔCFI = 0.004) showed good fit, which indicated that the Monitoring factor structure reached measurement invariance across the four samples. However, for the Blunting factor model, the model indices in the four samples showed poor fit, and the scalar invariance model was unacceptable (ΔCFI = 0.011). Therefore, measurement invariance of the Blunting sub-scale can not be full verified.

**Table 3:** model fit indices of the Monitoring factor model in the four samples

Sample group	CMIN/DF	GFI	AGFI	CFI	RMSEA	SRMR
Student	2.293	0.967	0.950	0.934	0.044	0.045
Medical staff	2.455	0.955	0.933	0.903	0.054	0.051
patient	1.029	0.902	0.853	0.989	0.018	0.079
Patient caregiver	1.132	0.907	0.856	0.948	0.037	0.070

*CMIN/DF* chi-square/degrees of freedom, *GFI* goodness-of-fit index, *AGFI* adjusted goodness of fit index, *CFI* comparative fit index, *RMSEA* root mean square error of approximation, *SRMR* standardized root mean square residual

### Floor and ceiling effects

**Fig.4:**
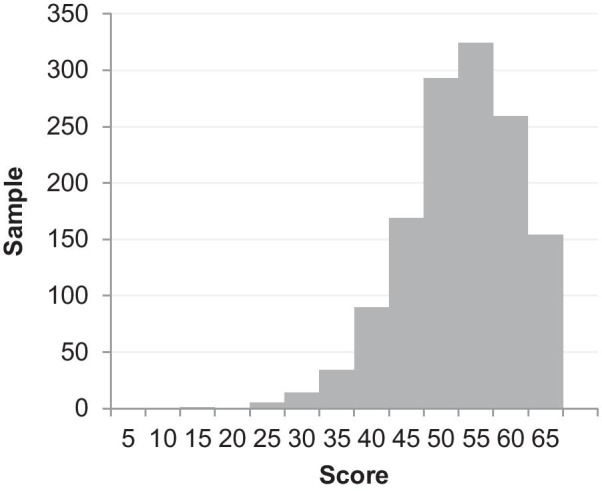
Score distribution of the Monitoring sub-scale

**Fig.5:**
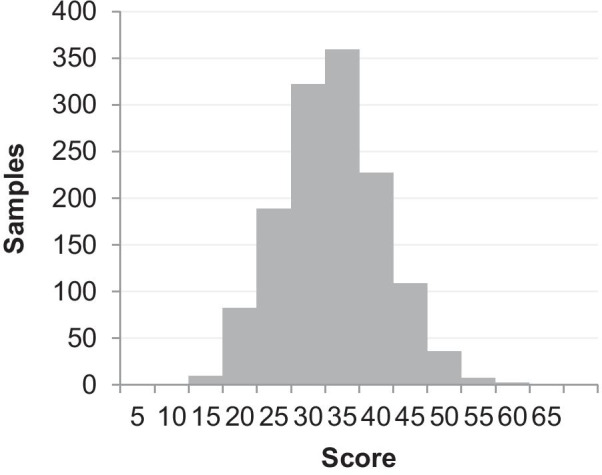
Score distribution of the Blunting sub-scale

Floor and ceiling effects of the two sub-scales were evaluated through the score distribution (Fig. 4 and Fig. 5). For Monitoring sub-scale, 0% got the lowest score (13) and 3% got the highest score (65); for Blunting sub-scale, 0.2% got the lowest score (13) and 0% got the highest score (65). Therefore, no floor and ceiling effects existed.

## Discussion

As a widely used tool to measure information-seeking styles, the MBSS has been translated and cross-culturally adapted into multiple languages among cancer populations, undergraduate students, surgical patients and normal adults, etc. [15, 20–22, 37, 38]. According to the cultural appropriateness, some modifications have been made in the MBSS. For example, The Spanish version of the MBSS kept 16 items which were close to their everyday experiences [21]. The German version of the MBSS added some controllable situations [37]. In our study, we made appropriate cultural adaption of the MBSS into Chinese version and deleted six items to form a 26-item C-MBSS. According to the experts, item 1, 18, 24, 25 and 26 were deleted resulting from unable to differentiate information-seeking styles in the context of Chinese culture. Ideas about Confucianism and collectivism are cherished and valued in Chinese culture. For the deletion of item 1, one plausible explanation is that Chinese patients are used to subordinate in the doctor-patient relationship and patient empowerment is not common [39]; therefore, most patients prefer to listen to the doctors rather than asking questions. Item 1 ‘I would ask the dentist exactly what work was going to be done.’ might not suit Chinese health care background. The Confucian principle of hierarchy and obedience emphasize power and social ranking in the organizations of Chinese society [40, 41], which means the inferiors are used to be appraised by their superiors instead of by themselves, hence item 18 ‘I would review the list of duties for my present job and try to figure out if I had fulfilled them all.’ seems inappropriate for Chinese population. In the face of being laid off, the loyal Chinese subordinates show their loyalty and dedication to the supervisors [40] and they will continue doing their work whatever happened; therefore, for item 24: ‘I would continue doing my work as if nothing special was happening.’, most of the people might choose to do so no matter what type of information-seeking styles they belong. From childhood, Chinese people are trained not to disobey [41], so both monitoring and blunting type person might choose to read the safety notice card as required by the crewman, and the item 25 ‘I would carefully read the information provided about safety features in the plane and make sure I knew where the emergency exits were’ might not identify the different information-seeking styles. The collectivist culture emphasis on people instead of task. For example, people would pause and chat with their friends when meeting them on the way to work in the collectivist culture [42]. For item 26 ‘I would make small talk with the passenger beside me’, both the monitoring and blunting type person might choose to do so because of cultural characteristic. Item 2 was deleted in the EFA process due to low factor loading. For item 2 ‘I would take a tranquilizer or have a drink before going.’, the reason for its deletion might be due to the fact that Chinese patients don’t have the habit of taking a tranquilizer or having a drink before seeing a doctor.

We performed EFA for four hypothetical stress-evoking scenarios of the C-MBSS separately and EFA of each scenario obtained a two-factor solution that explained 52.99%, 43.98%, 50.45% and 51.9% respectively of the variance in the research. The results of CFA demonstrated moderately model fit and provided confirmatory evidence for the factor structure. When we performed first-order analysis, we found strong correlations among the eight factors in the C-MBSS, which suggested high-order latent variants. The second-order CFA resulted in a 2-factor assumption (Monitoring and Blunting), and the values of T, CMIN/DF, GFI, AGFI, CFI, RMSEA and SRMR demonstrated the acceptable model fit and proved the feasible 2-factor solution. The results indicated moderately good construct validity for the C-MBSS, which is in consistent with other studies that the MBSS of different language versions had good or modest construct validity [12, 20, 21].

In our study, the Cronbach’s alpha coefficient for the Monitoring sub-scale was within acceptable limits (0.75), while the Blunting sub-scale had a lower Cronbach’s alpha coefficient (0.62). The results were similar with the literature review about the psychometric properties of the MBSS, which reported an average Cronbach’s alpha coefficient of 0.71 for the Monitoring sub-scale and 0.63 for the Blunting sub-scale [20]. In addition, it is reported by the original author and other researchers that the Monitoring sub-scale had the most utility with regard to predictive performance and it is often used independently to measure individual’s information-seeking styles [12, 15, 37].

As observed in this study, MGCFA results supported the measurement invariance for the Monitoring sub-scale but failed to verify the measurement invariance for the Blunting sub-scale across the four samples. Our study further confirmed Miller’s claim that the Monitoring sub-scales has better measurement performance and can be used across populations (i.e., patients, students, general population) [12]. Furthermore, no floor or ceiling effects were identified in the two sub-scales, which further demonstrated reliable content validity [30].

Giving the findings above, the Monitoring sub-scale of the C-MBSS can be used independently to measure individual’s information-seeking styles in Chinese contexts across different populations. It has been proved that health education matching individual’s information-seeking styles will lead to many positive health outcomes, oppositely, health education inconsistent with information-seeking styles is not conducive to patients’ well-being. Hence, the information-seeking styles of patients should be taken into consideration when assessing issues of information need and patient education [12]. As a valid tool to assess individual’s information-seeking styles, both the Monitoring and Blunting sub-scales of the MBSS have been widely used in patient education and counseling to improve patients’ health outcomes in other countries. In the randomized clinical trial conducted by Miller et al., they used the Monitoring sub-scale of the MBSS to assess individual’s information-seeking styles and indicated tailoring cervical cancer risk communication to monitoring attentional style may help improve adherence to follow-up recommendations after an abnormal Pap smear test result, which may contribute to decreased mortality from cervical cancer [15]. Williams-Piehota et al. evidenced that providing messages matched to information-seeking styles assessed and categorized by the abbreviated version of MBSS is an effective way for promoting mammography utilization, which may help reduce breast cancer mortality [43]. Sherman et al. showed that Monitoring processing style, which is assessed by the Monitoring sub-scale of MBSS, was found to predict post-surgical pain and suggested that extensive information about pain management for monitors may reduce post-surgical pain and improve health outcomes [19]. With the application of the Monitoring sub-scale of the C-MBSS, interventions can be designed to tailor patients’ information-seeking styles and improve the results of health education in Chinese population.

### Limitation

There are several limitations that should be taken into consideration. Although we have included a large and diverse sample size, the generalization of our findings might be limited due to the use of convenience sampling. The participants were mainly recruited from the southwest of China and unable to represent all people in China. In addition, due to practical constraints, we did not test the convergent/divergent validity and test–retest reliability of the C-MBSS. The convergent/divergent validity and test–retest reliability may be conducted in the future research.

## Conclusions

Our research examined the content validity, construct validity and internal consistency reliability of the C-MBSS among university students, medical staff, patients receiving percutaneous coronary intervention and their caregivers in China. We deleted six items due to weakness in differentiating information-seeking styles in the context of Chinese culture, resulting in a 26-items C-MBSS, which showed good content and construct validity. Compared with the Blunting sub-scale of the C-MBSS, the Monitoring sub-scale had acceptable internal consistency reliability, which is a reliable and valid instrument for identifying individual’s information-seeking styles among different population in China. By using the Monitoring sub-scale of the C-MBSS, medial staff can assess patients’ information-seeking styles, and give them the information that is tailored to their information-seeking styles, which can help individuals fare better (psychologically, behaviorally, and physically), and enhance the effectiveness of health education in the end.

## Data Availability

The datasets used and/or analyzed during the current study are available from the corresponding author on reasonable request.

## Abbreviations

MBSS: Miller Behavioral Style Scale
C-MBSS: Chinese Version of Miller Behavioral Scale
MGCFA: Multigroup confirmatory factor analysis
I-CVI: Item-Level Content Validity Index
S-CVI/Ave: Averaging Scale-Level Content Validity Index
EFA: Exploratory factor analysis
CFA: Confirmatory factor analysis
AMOS: Analysis of moment structure
CMIN/DF: Chi-square/degrees of freedom
GFI: Goodness-of-fit index
AGFI: Adjusted goodness of fit index
CFI: Comparative fit index
RMSEA: Root mean square error of approximation
SRMR: Standardized root mean square residual
